# Metreleptin for the treatment of progressive encephalopathy with/without lipodystrophy (PELD) in a child with progressive myoclonic epilepsy: a case report

**DOI:** 10.1186/s13052-020-00916-2

**Published:** 2020-10-24

**Authors:** Stefania Pedicelli, Luca de Palma, Caterina Pelosini, Marco Cappa

**Affiliations:** 1 Unit of Endocrinology, Bambino Gesù Children’s Hospital, Rome, Italy; 2 Rare and Complex Epilepsy, Department of Neuroscience, Bambino Gesù Children Hospital, Rome, Italy; 3 Chemistry and Endocrinology Laboratory, University Hospital of Pisa, Pisa, Italy

**Keywords:** Case report, Congenital generalized lipodystrophy type 2, Metreleptin, Neurological impairment, Progressive myoclonic epilepsy

## Abstract

**Background:**

A number of genetic syndromes associated with variants in the *BSCL2*/seipin gene have been identified. Variants that cause skipping of exon 7 are associated with progressive encephalopathy with/without lipodystrophy (PELD), which is characterized by the development of progressive myoclonic epilepsy at a young age, severe progressive neurological impairment, and early death, often in childhood. Because the genetic basis of PELD is similar to that of congenital lipodystrophy type 2, we hypothesized that a patient with PELD may respond to treatments approved for other congenital lipodystrophic syndromes.

**Case presentation:**

We describe a 5-year-old boy with an extremely rare phenotype involving severe progressive myoclonic epilepsy who received metreleptin (a recombinant analogue of leptin) to control metabolic abnormalities. At the age of two, he had no subcutaneous adipose tissue, with hypertriglyceridemia, hypertransaminasemia and hepatic steatosis. He also had a moderate psychomotor delay and generalized tonic seizures. At 4 years, he had insulin resistance, hypercholesterolemia, hypertriglyceridemia, mild hepatosplenomegaly and mild hepatic steatosis; he began a hypolipidemic diet. Severe psychomotor delay and myoclonic/myoclonic atonic seizures with absences was evident. At 5 years of age, metreleptin 0.06 mg/kg/day was initiated; after 2 months, the patient’s lipid profile improved and insulin resistance resolved. After 1 year of treatment, hepatic steatosis improved and abdominal ultrasound showed only mild hepatomegaly. Seizure frequency decreased but was not eliminated during metreleptin therapy.

**Conclusions:**

Metreleptin may be used to control metabolic disturbances and may lead to better seizure control in children with PELD.

## Background

Lipodystrophy syndromes are a group of rare, highly heterogeneous disorders characterized by generalized or partial lack of adipose tissue and deficiency of the adipocyte-secreted hormone leptin [1–4]. Lipodystrophies are inherited or acquired, and are classified into four major subtypes: congenital or acquired generalized lipodystrophy, and familial or acquired partial lipodystrophy [2, 3, 5]. Multiple congenital lipodystrophy phenotypes have been identified, each with distinct clinical features [5–7]. Conditions associated with variants in the *BSCL2*/seipin gene include progressive encephalopathy with/without lipodystrophy (PELD), also called Celia’s encephalopathy (MIM#615924) and congenital general lipodystrophy type 2 (CGL2) [2, 5]. PELD is associated with *BSCL2* variants that cause skipping of exon 7 and is characterized by the development of progressive myoclonic epilepsy (PME) at a young age and severe progressive neurological impairment, which is rapidly fatal in many, but not all, cases [8–14].

Without effective treatment, the prognosis for patients with PELD is poor because of the intractable epilepsy and rapid neurodegeneration [10, 11, 13]. Preliminary data suggest that leptin replacement therapy, combined with dietary intervention, may provide some benefit in patients with PELD [9]. Metreleptin, a recombinant analogue of leptin, has been shown to improve metabolic abnormalities in patients with lipodystrophy, including pediatric patients with CGL2, and is currently the only drug indicated specifically for the treatment of generalized lipodystrophy [2, 6, 15]. However, due to the extremely rare nature and particularly poor prognosis of many congenital lipodystrophies, little is known about the utility of metreleptin in affected children with severe neurological involvement [9]. Here, we report the effects of metreleptin therapy on the metabolic and neurological complications of PELD in a child with PME.

## Case presentation

The male patient was born at term by natural delivery to non-consanguineous parents in Macedonia in 2013, following a normal pregnancy. In the first month of life, he presented with generalized hypotonia. At 12 months, he developed monthly tonic seizures, which were well-controlled with valproic acid.

At 2 years, the patient moved to Italy and received new medical evaluation. His height was >97th percentile and his body mass index (BMI) was ~10th percentile, with an almost complete absence of subcutaneous adipose tissue. Metabolic testing revealed hypertriglyceridemia, hypertransaminasemia and hepatic steatosis so a low-fat diet was implemented. The patient also had a moderate psychomotor delay; seizures became more frequent, characterized by absences with myoclonic/myoclonic atonic phenomena. Seizures were pharmacoresistant. Electroencephalogram (EEG) showed generalized epileptic abnormalities, while brain magnetic resonance imaging was normal. Molecular analysis of the *BSCL2* gene revealed a homozygous c.(1076dupC)/p.(Glu360*) variant.

At 4 years, the patient had hypercholesterolemia and hypertriglyceridemia, with partial improvement of cholesterol and triglyceride levels after 3 months of hypolipidemic nutrition (Table 1). Oral glucose tolerance testing (OGTT) at 1.75 mg/kg revealed normal glucose tolerance (baseline: 75 mg/dL, 2 h: 82 mg/dL), although the homeostasis model assessment of insulin resistance (HOMA-IR) value was high (2.6). The glycosylated hemoglobin (HbA1c) level was 29 mmol/mol. Abdominal ultrasound revealed mild hepatosplenomegaly with increased liver echogenicity related to mild steatosis; echocardiography revealed mild left ventricular hypertrophy with normal function. His neurological condition continued to deteriorate: he was able to walk but was frequently falling due to his seizures, he had severe intellectual disability, and the EEG showed a progressive slowing of the background activity with generalized spike and wave associated with myoclonic, myoclonic-atonic seizures. He required hospitalization on several occasions due to prolonged seizures. Therefore, a vagal nerve stimulator was implanted with a rapid (1 month) up-titration of the stimulation parameters. Within that month, the patient experienced a consistent reduction in seizure frequency; while he was still experiencing up to 20 seizures/day he had no more episodes of status epilepticus. The detailed neurological manifestations of this case have previously been reported [16]. Four months after implantation, the seizures reappeared, with a transient deterioration of EEG background activity, and perampanel was initiated. Daily seizures (up to 60 seizures/day) persisted, but the patient had no episodes of status epilepticus.

**Table 1:** Fasting lipid profile before and after starting treatment with metreleptin 0.06 mg/kg/day

Lipid levels (mg/dL)	Age 4 years (no metreleptin)		Age 5 years (baseline before starting metreleptin)	Age 5 years (2 months after starting metreleptin)
	Pre-diet	Post- diet		
Cholesterol				
Total	212	117	129	102
HDL	33	30	29	39
LDL	100	74	74	51
Triglycerides	396	102	121	60

*HDL* high-density lipoprotein, *LDL* low-density lipoprotein

When he was 5 years old (10 months after perampanel was initiated), he was hospitalized with new episodes of prolonged seizures with mixed tonic and myoclonic components. BMI was at the 10th percentile. Subcutaneous metreleptin 0.06 mg/kg/day was initiated and was well tolerated. After 2 months’ therapy, his overall lipid profile improved (Table 1). OGTT was normal (baseline: 59 mg/dL, 2 h: 75 mg/dL) and HOMA-IR had decreased to 0.33. The HbA1c was static at 29 mmol/mol; neurological status was stable and without prolonged seizures. Serum leptin level (0.4 ng/mL) was normal-to-low for his BMI (0.25–3.20 ng/mL) prior to metreleptin; after 3 month’s therapy, leptin was 0.4 ng/mL before and 10.8 ng/mL after the daily dose.

After 1 year of treatment with metreleptin, HOMA-IR had increased to 6.02, but due to a lack of supply the patient had not been receiving therapy for the preceding month. Abdominal ultrasound showed mild hepatomegaly and an improvement in hepatic steatosis. The patient had grown 8.13 cm/year and his BMI was below the third percentile.

Adherence to therapy during treatment was ensured by a direct hospital supply of medication. No adverse effects have been reported or found during follow-up, including from results of blood examinations, ultrasound, and clinical evaluation. In the subsequent 2 years, he did not experience any prolonged epileptic seizure leading to hospitalization. He still experienced 2–3 seizures/month, which resulted in sudden falling down, and daily (up to 30 seizures/day) myoclonic seizures involving mainly the perioral muscles. An EEG did not show any evidence of neurological modification from the pre-treatment period.

## Discussion and conclusions

We describe improvements in insulin sensitivity, lipid levels and hepatic steatosis with metreleptin 0.06 mg/kg/day in a child with a homozygous c.1076dupC *BSCL2* gene variant, which had resulted in generalized lipodystrophy with PME and severe progressive neurological impairment.

The metabolic results observed in our patient were similar to those demonstrated by Brown et al. in pediatric patients with different types of lipodystrophy [15]. In their report, metreleptin treatment outcomes both in the short (1 year) and long term (mean 5 years) were better in adolescents (aged 12–18 years) than in children; this result is probably influenced by the small number of the children patients included in the study [15]. The clinical evidence to date confirms studies in animal models – leptin-treated ob/ob mice and n-SREBP-1c lipodystrophic mice – that showed leptin’s hypoglycemic food intake-independent effect, attributable to improvements in peripheral insulin sensitivity [17–20]. Even though the exact mechanism of action of metreleptin has not yet been fully elucidated, metreleptin seems to contribute to body weight loss by improving insulin sensitivity rather than increasing energy expenditure.

Metreleptin-associated improvements in the lipid profile of patients may be mediated by increased expression of enzymes and transcription factors involved in fatty acid oxidation and decreased expression of those regulating fatty acid synthesis, as demonstrated in rodent models [21–23].

We previously described the neurological condition of our patient [16] before metreleptin treatment. After treatment, we saw a clear benefit from an epileptogenic point of view, with a reduction in seizure burden and a drop in hospitalizations. The neurological impairment remains stable. These results are in line with a previous case reported by Araújo-Vilar and colleagues, who demonstrated a clear slowdown in neurological regression with metreleptin treatment in combination with a diet rich in polyunsaturated fatty acids in a child with PELD [9].

It should be noted that, as in-depth histological evidence of neuronal inclusion and molecular studies were not carried out, we cannot rule out the possibility that this child had a variant of CGL2, and not PELD. However, in a previous report of similar cases, Opri and colleagues reported two cases of CGL2 that were due to variant c.974dupG in *BSCL2* [11]. Further research by Sanchez-Iglesias and colleagues showed that this is the same mechanism as the c.985C > T variant in *BSCL2* that causes PELD [13]. Moreover, this PELD variant leads to the same adipose and neurological phenotypes as described in the original report by Opri and colleagues, leading Sanchez-Iglesias et al. to conclude that the patients reported by Opri et al. actually had PELD and not CGL2 [13].

This case report contributes to the evidence that certain *BSCL2* variants are linked with the rare condition PELD, which is characterized by PME and progressive neurodegeneration, and that metreleptin should be used to control metabolic disturbances in children with this syndrome. Metreleptin may have dual benefits in these children, effectively treating metabolic abnormalities, as observed in our patient, and allowing better seizure control, even if our patient had a relatively short-term follow-up.

## Data Availability

Data sharing is not applicable to this article as no datasets were generated or analyzed during the current study.

## Abbreviations

BMI: Body Mass Index
CGL: Congenital Lipodystrophy
EEG: Electroencephalogram
HbA1c: Glycosylated Hemoglobin
HOMA-IR: Homeostasis Model Assessment Of Insulin Resistance
OGTT: Oral Glucose Tolerance Test
PME: Progressive Myoclonic Epilepsy
